# Identification and prevention of heterotopias in mouse neocortical neural cell migration incurred by surgical damages during utero electroporation procedures

**DOI:** 10.1080/19768354.2020.1737225

**Published:** 2020-03-09

**Authors:** Bolin Wang, Liting Ji, Kausik Bishayee, Changyu Li, Sung-Oh Huh

**Affiliations:** a School of Pharmacy, Zhejiang Chinese Medical University, Hangzhou, People’s Republic of China; b Department of Pharmacology, College of Medicine, Institute of Natural Medicine, Hallym University,Chuncheon, South Korea

**Keywords:** *In utero* electroporation, micro-injection, artificial heterotopias and ectopias, layer marker Ctip2 and TBR1

## Abstract

*In utero* electroporation (IUE) is a useful technique for gene delivery in embryonic mouse brain. IUE technique is used to investigate the mammalian brain development *in vivo*. However, according to recent studies, IUE methodology has some limitations like the formation of artificial ectopias and heterotopias at the micro-injection site. Thus far, the artificial heterotopias generated by physical trauma during IUE are rarely reported. Here, we reported the artificial heterotopias and ectopias generated from surgical damages of micropipette in detail, and moreover, we described the protocol to avoid these phenotypes. For the experimental purpose, we transferred empty plasmids (pCAGIG-GFP) with green fluorescent-labelled protein into the cortical cortex by IUE and then compared the structure of the cortex region between the injected and un-injected cerebral hemispheres. The coronary section showed that ectopias and heterotopias were appeared on imperfect-injected brains, and layer maker staining, which including Ctip2 and TBR1 and laminin, can differentiate the physical damage, revealing the neurons in artificial ectopic and heterotopic area were not properly arranged. Moreover, premature differentiation of neurons in ectopias and heterotopias were observed. To avoid heterotopias and ectopias, we carefully manipulated the method of IUE application. Thus, this study might be helpful for the *in utero* electroporator to distinguish the artificial ectopias and heterotopias that caused by the physical injury by microneedle and the ways to avoid those undesirable circumstances.

## Introduction

Neuronal disorders have a significant impact on the newborns, faulty genetic distribution can contribute to different disease related to brain development. Therefore, it is important to understand the role of a specific gene during the neuro-development process. Therefore, gene manipulation in the embryonic mouse brain is important for understanding how the genetic disturbances contribute to either disease etiology or developmental disorders.

*In utero* electroporation (IUE) is a useful technique for gene manipulation in a particular part of the brain (Nishimura et al. 2012; Hong et al. 2018; Hu et al. 2018; Kim et al. 2018). The expression vectors are microinjected into the embryonic brain ventricle, and transfect the neuronal precursor cells with the DNA by square-wave electric pulses (Niwa et al. 2010; Youngpearse et al. 2010). Using this technique, the DNA can be delivered or transfected into the cerebral cortex, diencephalon, hindbrain, and spinal cord region (David et al. 2014; Nicole et al. 2018; Watanabe et al. 2018) and used for the evaluation of the phenotypic changes that are relevant to the disease pathology (Cwetsch et al. 2018). But IUE technique has different drawbacks that could lead to different unexpected false or imitation results, such as surgical damages to cause artificial ectopias and heterotopias. In this paper, we will discuss different issues with IUE during the injection procedure.

Traditional gene manipulation techniques like gene-targeting technologies using embryonic stem (ES) cells, zinc-finger nucleases (ZFNs) technology and transcription activator-like effector nucleases (TALENs) technology were used to study neuro-development from ages (Pavletich and Pabo 1991; Li et al. 1992; Capecchi 2005; Moscou and Bogdanove 2009; Higashijima et al. 2017; Boch et al. 2009). The major disadvantages of these methods are such as limitation in transfection efficiency and specio-temporal gene manipulation, anduncontrolled or non-specified transfection. IUE has several advantages over the traditional techniques like higher transfection efficiency, lower cytotoxicity, time-efficient, and multiple plasmids can be simultaneously transfected into the same cells (Saito 2006; Cwetsch et al. 2018). IUE requires neither complex surgery nor stereotaxic apparatus, so now, IUE is widely used to understand the mechanism of brain development (Carrel et al. 2015; Broix et al. 2018). The detail of the advantages of IUE is described in Table 1.

**Table 1.** Advantages of *in utero* electroporation

Advantages	Details	References
High efficiency, low cytotoxicity	No significant increase in cell death has been detected after electroporation	Saito (2006), Mizutani and Saito (2005)
Simple and quick procedure	*In vivo* electroporation for gene transfer into more than 10 embryos can be carried out within 30 min	Saito (2006)
Multiple gene targeting	Multiple genes on different plasmids can be simultaneously transfected into the same cells	Saito and Nakatsuji (2001)
Region and time-specific gene targeting	Only the side of the ventricle that is close to the anode is transfected	Saito and Nakatsuji (2001), Saba et al. (2003), Cwetsch et al. (2018)

However, in IUE, improper injection method can obtain an unwanted or false outcome that can confuse researchers. Therefore, every result obtained by IUE should carefully be evaluated for the formation of pseudo-heterotopia or pseudo-ectopia-like structures. Heterotopia is clump-like structures in the ventricular or periventricular zone and forms due to faulty migration of the neurons during development (Ishii et al. 2015; Manent et al. 2009). The similar structural deformities could be formed by injury from the mishandling of the needle. The first instance pseudo-heterotopia was documented by Rosen et al., in 1995, he induced heterotopias and ectopias in the cerebral cortex of newborn mice through inserting a hypodermic needle into developing cerebral cortex (Rosen et al. 1995). Micropipette needle can also induce physical trauma on the cortex which would disturb the process of neuronal migration and can result in pseudo-heterotopia and pseudo-ectopia-like structures.

In this article, we described different aspects of IUE that can cause unnatural outcomes. While the artificial heterotopias generated by surgical procedures in IUE are rarely reported until now. Here, we report the artificial heterotopias generated from physical injuries by micropipette in detail and described the measures to avoid the artificial heterotopias. We performed IUE with pCAGIG-GFP plasmid to identify the transfected neurons in the ventricles. Later, the cortex was stained with different cellular markers like Tuj1 (neuronal differentiation marker) (Halbach 2007), Ctip2 (cortex layer 5 and 6 marker) (Leyva and López 2013), TBR1 (cortex deep-layer marker) (Englund et al. 2005), Laminin (blood vessels in brain marker) (Xu et al. 2019), Nestin (progenitor and radial glia (RG) marker) (Halbach 2007), Reelin (telencephalon marginal zone marker) (Goffinet 2017), KI-67 (proliferation marker) (Menon et al. 2019), BrdU (proliferation and DNA synthesis marker) (Halbach 2007) to identify and understand the defects forms due to surgical damages during IUE.

## Materials and methods

### Animals and housing

Pregnant mice of C57BL/6N strain were used in this study for IUE. The mice were purchased from Orient Bio (Seongnam, South Korea). All the experiments were approved by the Institutional Animal Care and Use Committee (IACUC) of Hallym University, South Korea (approval number: Hallym 2015-7). Fetuses at E13.5–E17.5 were used for the experimental purpose. According to Orient Bio’s mating schedule, the vaginal plug at noon of the day was defined as embryonic day 0.5 (E0.5). The mice were housed in optimum conditions (12 h light/day cycle with 22 ± 2°C and 50 ± 10% humidity) and mice feeds were procured from a commercial food company (Purina Inc., Seongnam, Korea).

### Preparation of plasmid DNA glass capillary

The pCAGIG plasmid vector containing ampicillin selection marker were grown in *Escherichia coli*- DH5a strain. The plasmid DNA was isolated from Luria Bertani (LB) broth culture with ampicillin by using Qiagen EndoFree^®^ Plasmid Maxi Kit. DH5a strain was grown in standard LB medium to a cell density of ∼3–4 × 10^9^ cells/ml. DNA was extracted by following the manufacturer’s protocol and the DNA was precipitated by isopropanol to the eluted DNA. Mix and centrifuge immediately at 15,000 g for 30 min at 4°C and the eluted DNA was washed with endotoxin-free 70% ethanol at room temperature. Before the *in vivo* electroporation, the DNA was dilated to a final concentration of 2 µg/µl in 0.1% fast green (Sigma) solution and TE buffer (10 mM Tris base, 1 mM EDTA solution, pH 8.0). G1 glass capillary (Narishige Scientific Instrument Lab, Tokyo, Japan) was used for the injection. The sharp end on the glass capillary for the injection was made using a micropipette puller (Narishige, Model-PC10). Then the capillary end was pinched off with forceps to make the tip with 20–30 µm diameter.

### Preparation for surgery

Pregnant mice were anesthetized using Isoflurane – Ifran (Hana Pharm. Co. Ltd., Kyonggi-do, Korea) with a constant supply of oxygen and nitrogen gas mixture. The hair from the abdomen was removed by using a razor blade and 83% molecular grade ethanol, later the shaved skin region was sponged with the povidone-iodine topical solution to remove any contamination. The incision was made at the abdominal midline with fine scissors and then all uterine horns were carefully pulled out onto a 37°C sterilized pre-warmed phosphate-buffered saline (PBS)-moistened autoclaved cotton gauze, the uterus was kept moist with PBS during the surgical period.

### *In utero* electroporation

The expression vector-pCAGIG containing green fluorescent protein (GFP) was used for electroporation. Pregnant mice of different stages were anesthetized using 4% isoflurane. After surgery, plasmid DNA (2 mg/ml) in PBS containing 0.01% fast green stain (Sigma–Aldrich, St. Louis, USA) was microinjected through the uterine wall into the lateral ventricles of target embryos. Using Tweezertrodes (5 mm diameter) (BTX/Harvard Biosciences, Holliston, USA) across the embryo’s brain through the uterus, electroporation was performed by discharging 5 pluses of the 45 V for 50 ms duration and 950 ms interval. The intact uterus was then put back to the abdomen and the abdominal cut was carefully and aseptically sutured back. The mice were allowed to recover in pre-warmed cages. The electroporated embryos were then allowed to develop in the pregnant mice. The transfected GFP-positive embryo brains were shorted for the further experimental process.

### Immunohistochemistry and identification of ectopias and heterotopia

The extracted brain or whole skull, depending on the embryonic stage, immersed in 4% paraformaldehyde (PFA) at 4°C for 2 h for fixation. Then the brains were stored in PBS containing 30% sucrose at 4°C-overnight for the hardening process. Later, the brains were washed with OCT solution to remove the excess sucrose–PBS solution and embedded in OCT. The coronal sections (10 µm) from brains were sliced using the cryo-section machine.

For immunostaining, the antigen retrieval process was performed by heating the slides in citrate buffer (10 mM, pH 6.0) at 95°C for 5–10 min. The samples were blocked in 5% donkey serum in PBS plus 0.1% Triton-X100 (PBST) solution. The sections were subsequently incubated with anti-BrdU (Jackson Immunorearch) (1:400), anti-KI67 (Abcam) (1:100), anti-GFP (Abcam) (1:100), anti-Tuj1, anti-Ctip2 (Abcam) (1:500), anti-TBR1 (Abcam) (1:500), anti-Laminin (Sigma) (1:1000), anti-Nestin (DSHB) (1:400), and anti-Reelin (DSHB) (1:400) for overnight at 4°C in a humid chamber. Afterwards treatment with secondary antibody (conjugated with fluorescence) was carried out for 2 h at room temperature. A total of 15 embryos were used for the study of differentiation and proliferation staining. Fluorescent images were acquired with a laser-scanning confocal microscope (LSM710) (Zeiss, Oberkochen, Germany).

## Result

### Formation of ectopias and heterotopias at micropipette puncture region at the micro-injection site

**Figure 1.**
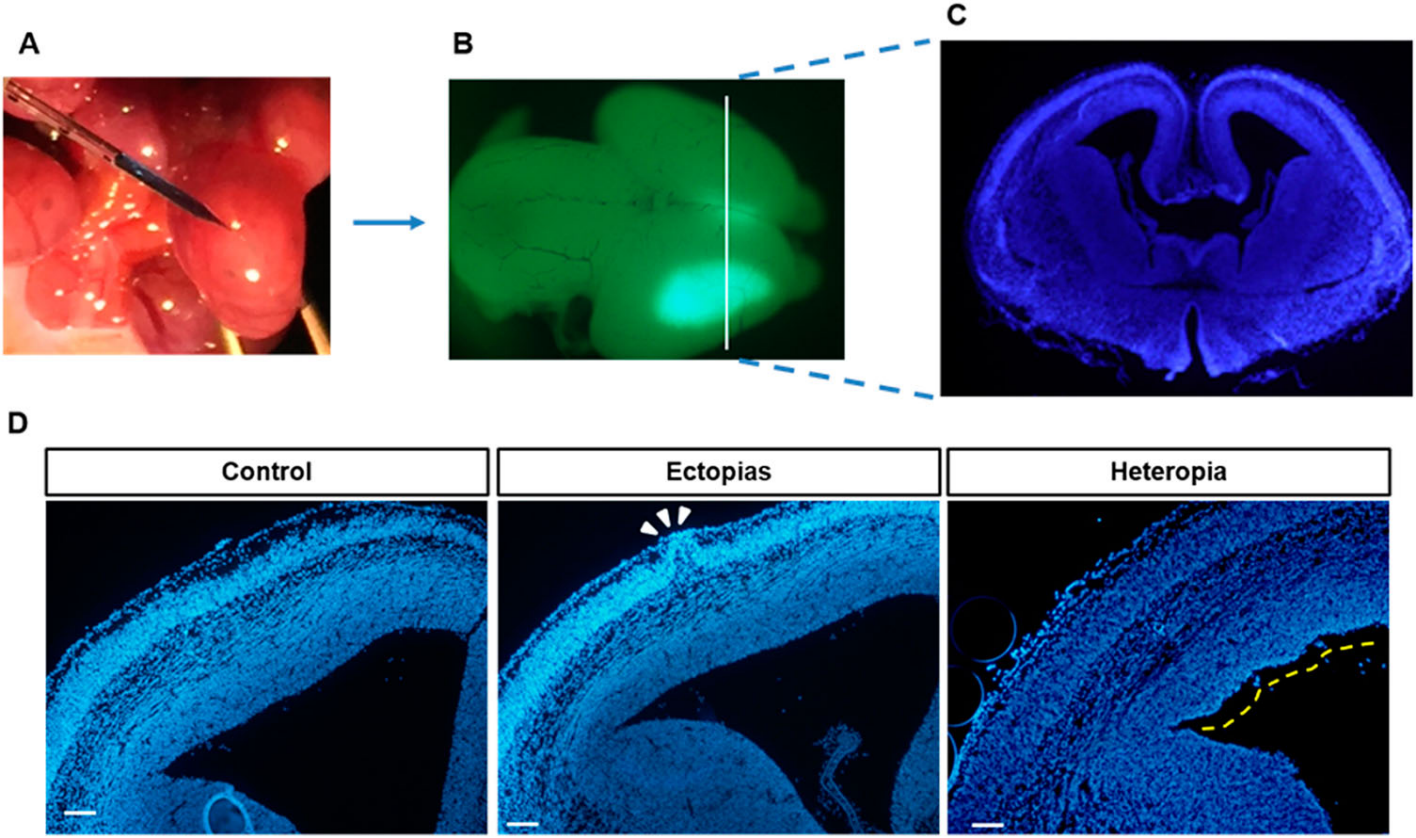
*In utero* electroporation with green-plasmid and identification of ectopias and heterotopias. (A) The embryonic mouse brains at E13.5 stage were injected with GFP-conjugated plasmid (pCAGIG) in 0.1% fast green solution and then transfected by electroporation. (B) The pCAGIG electroporated brains were harvested at E15.5 stage (48 h post-electroporation) and examined under Research Macro Zoom fluorescence system (Olympus DP72, Kanazawa-shi, Japan) for green fluorescence. (C) The rostral part of a coronal section from the injected brain was sectioned and then stained with DAPI (indicated white line from B was the magnified section that represents in C). (D) The harvested brains were sectioned and stained with DAPI to identify the Ectopia and Heterotopias. The Ectopia was indicated with white arrowheads and heterotopias were indicated by a yellow dashed line. Scale bar of the image is 100 μm.

To visualize the ectopia and heterotopia formation by *in utero* injection, the GFP-positive injected brains were sectioned (Figure 1(A–C)). The neuronal cells are stained with green color and nucleus was stained with blue color. The heterotopia-like lump structure was identified in low magnified brain slices and marked with a white arrow (Figure 1(D)). On the other hand, we also identified ectopia-like structures in the injected brains and marked with a yellow line, but in the control set, these types of structures were absent (Figure 1(D)). The improperly injected brains showed pseudo-ectopia and heterotopia-like lumps, but the lump-like structures was absent in the properly handled control set, and the ectopias and heterotopias in ventricles may result from physical injury by micropipette instead of the specific plasmid.

### Induction of premature differentiation at micropipette puncture region

**Figure 2.**
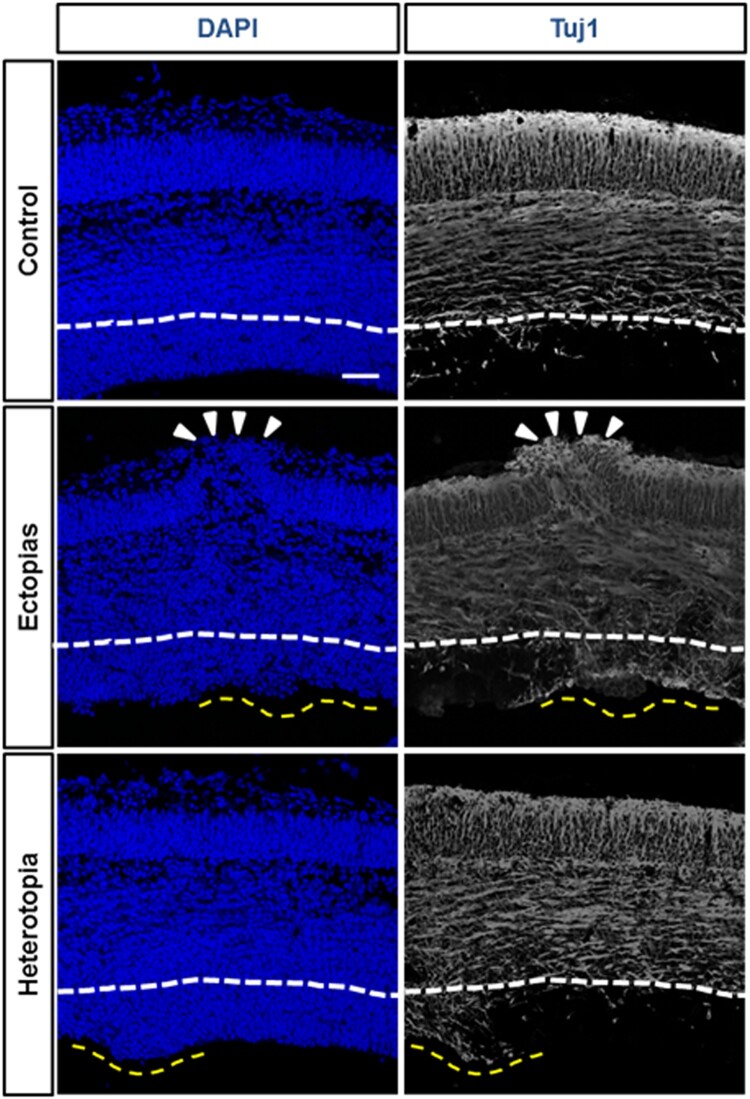
Differentiation status of neurons at the ectopic and heterotopic region. The embryonic mouse brains at E13.5 stage were injected GFP-conjugated plasmids and harvested at E 15.5 stage. DAPI (blue) and Tuj1 (white) staining were performed to stain nucleus and differentiated neurons. The ectopia was marked white arrowheads and heterotopias were by a yellow dashed line. The white line was marked for the boundary layer of undifferentiated neuronal region as per the control set. The Tuj1-positive differentiated neuronal population was markedly increased in the ectopic and heterotopic cortical section. The scale bar was 50 μm.

To understand the molecular etiology of the pseudo-ectopic or heterotopic mouse brain, we stained them with β-III-tubulin (Tuj1), a neuron-specific differentiation marker. In the control mouse brain, the ventricular zone (VZ) region was Tuj1-negative, suggesting that the progenitor cells were in dividing the state. But in heterotopias in VZ, we found that the region was Tuj1-positive, implying that the induction of premature differentiation of the neurons in the proliferative zone (Figure 2). The induction of early or premature differentiation could be one marker for the micropipette-mediated damage.

### Identification of damage cortex at micropipette puncture region

**Figure 3.**
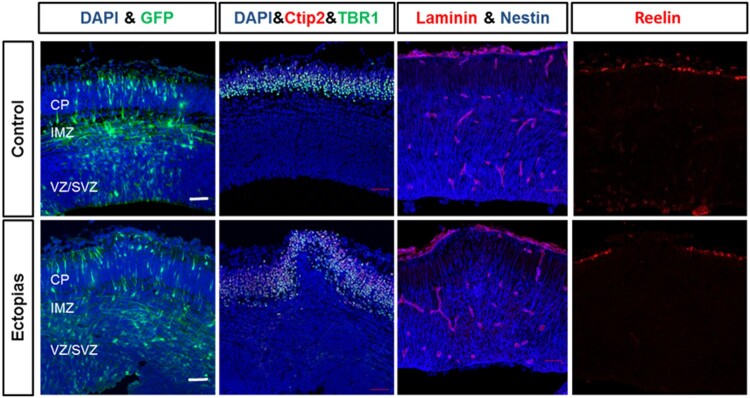
Deformation of cortical layers in section cortex. The embryonic mouse brains at E13.5 stage were injected GFP-conjugated plasmids and harvested at E15.5 stage. (First panel) The neuronal migration was observed by GFP staining (green) in control and ectopic cortical sections. (Second panel) The transfected brain sections were stained with layer markers like Ctip2 (red) and TBR1 (green) and counterstained with the nuclear stain DAPI (blue). (Third panel) The transfected brain sections were stained with basal lamina marker laminin (red) and radial glial cell marker nestin (blue). (Fourth panel) The transfected brain sections were stained with telencephalon marginal zone marker Reelin (red). The scale bar was 50 μm.

It is necessary to identify the damage cortex region of the developing brain by the micropipette puncture. In the control set, the GFP-positive neurons are migrated into the intermediate zone (IMZ) and cortical plate (CP) region, the neuronal morphological transition at IMZ was prominent. Alternatively, in the ectopic cortex region, the distribution of migrating neurons at IMZ and CP region was not as uniform as the control brains and observed morphological transition at IMZ was minimum (Figure 3). Therefore, to understand the distribution of the neurons at the cortex, we stained the cortex regions with different layer markers. First, we stained with ctip2 and TBR1 to stain different layers. In the control brain cortex, both the layers were distributed evenly as an indicator of normal cortical structure, but in the ectopic brain, at the micropipette puncture region, the layer pattern was changed into a convex curve pattern (Figure 3). The disruption of the layers was caused by the microneedle fractures.

Later, we stained the cortex with laminin and nestin which stains basal lamina and redial glial cells, respectively. The Laminin staining of the basal lamina of CP revealed that the control set has an intact and smooth structure, whereas that of the micropipette puncture region was of the convex structure and was broken also. We stained the RG cells with nestin, the radial glial cells in control extend long processes that terminate with their endfeet at the pial basement membrane, while the radial glial cells in micropipette puncture region arranged irregularly and bulged out of the pial surface (Figure 3).

To identify the rupture of the telencephalic marginal zone, we stained the cortex with reelin. Reelin stains the outer marginal layer of the cortex. In the control brains, the reelin stained a uniform outer layer but with ectopia, reelin staining showed the same tendency that neurons and other cells disrupt the reelin and over migrate out of the pial surface (Figure 3).

**Figure 4.**
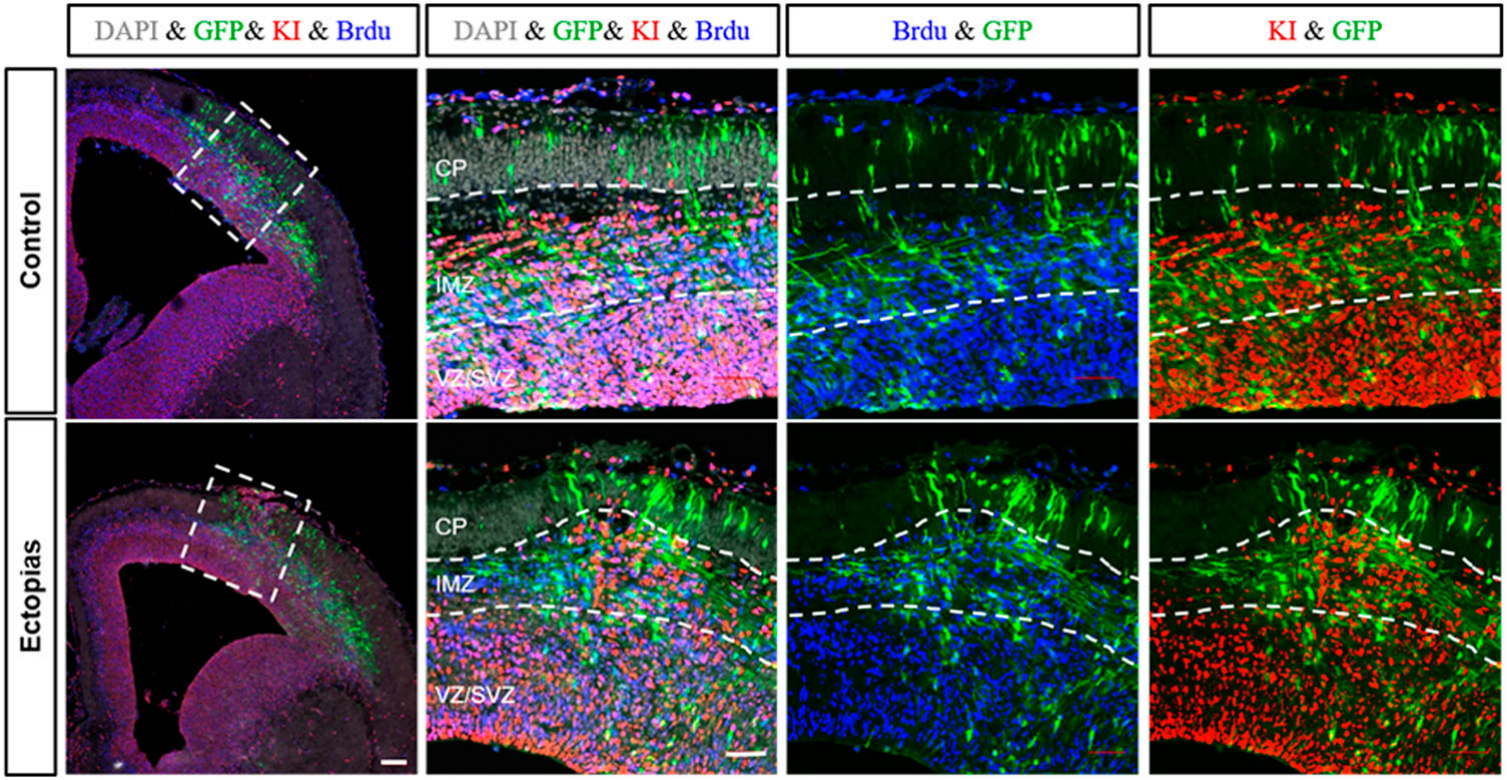
Proliferation status of neurons in the ectopic region. The embryonic mouse brains of E13.5 stage were injected GFP-conjugated plasmids and harvested at E15.5 stage. The cortical slices were stained with antibody for GFP (green), KI67 (red), BrdU (blue) and DAPI (gray). GFP-positive neurons at the ectopic site were over-migrated when compared with control section. KI67 and BrdU-positive neurons were found at the higher IMZ region of the ectopic cortex but were absent in control. White dashed lines were used to marked different zones of the cortex (CP, IMZ, and VZ/SVZ). The scale bars were 100 μm for the first panel and 50 μm for rest of the panels.

To identify whether the ectopic brains were consisting of proliferating cells, we stained the cortex samples with two proliferation markers, BrdU and KI67. In the control tissue, the proliferating cells were at IMZ and ventricular zone/subventricular zone (VZ/SVZ) area. While comparing with the ectopic brains, there were no significant changes in the number of proliferating cells, but the structure of the proliferating zone was changed with bulged out structure. There was a significant increase in the number of KI67-positive cells in the upper layer region in the ectopic brains, implying the leakiness or disruption of the neuronal layers (Figure 4).

### The solution to avoid artificial ectopias and heterotopias

In this section, we will be discussing the solutions to avoid the formation of ectopias and heterotopias in the cortical region of the developing brain.

#### (a) Precautions to follow for cortical injections to avoid artificial ectopias and heterotopias

Different precautious steps need to be followed during micro-injection in the embryo brain to avoid artificial ectopias and heterotopias.

- The glass micropipettes quality is essential to reduce artificial ectopia and heterotopia formation.
- The uterus needs to be held in a way that ventricles could be visualized easily. For proper injection, this step is important for handling the embryos and for identification of the proper injecting site.
- When the embryo was set up to be perfectly oriented for micro-injection, then it needs to be pierced the uterine wall with glass micropipette at an angle of 45° to the uterine wall.
- It is important to identify the rostral end of the gap between cortical hemispheres and the injection penetration should be of ∼1 mm.
- One microlitre of DNA-fast green solution needs to be injected into the ventricle before placing the electric pulse. The proper injection fills the ventricles with a blue color solution. The optimum pulse rate is described in Table 2 for plasmid DNA transfection.

**Table 2.** Optimal voltages for different embryonic stages of *in vivo* electroporation

Embryonic stage	Voltage (V)	Surviving embryos (%)	GFP+ embryos (%)
E12.5	35	>95	98
E12.5	45	< 10	–
E13.5	45	>95	>98
E14.5	45	∼100	>98
E15.5	45	∼100	>98
E16.5	45	∼100	>98

NB: E12.5–E14.5 embryos were generally used for cortical injection; and E14.5–E16.5 embryos for hippocampal injection.

#### (b) Formation of hippocampus and identification of suitable stage for IUE

**Figure 5.**
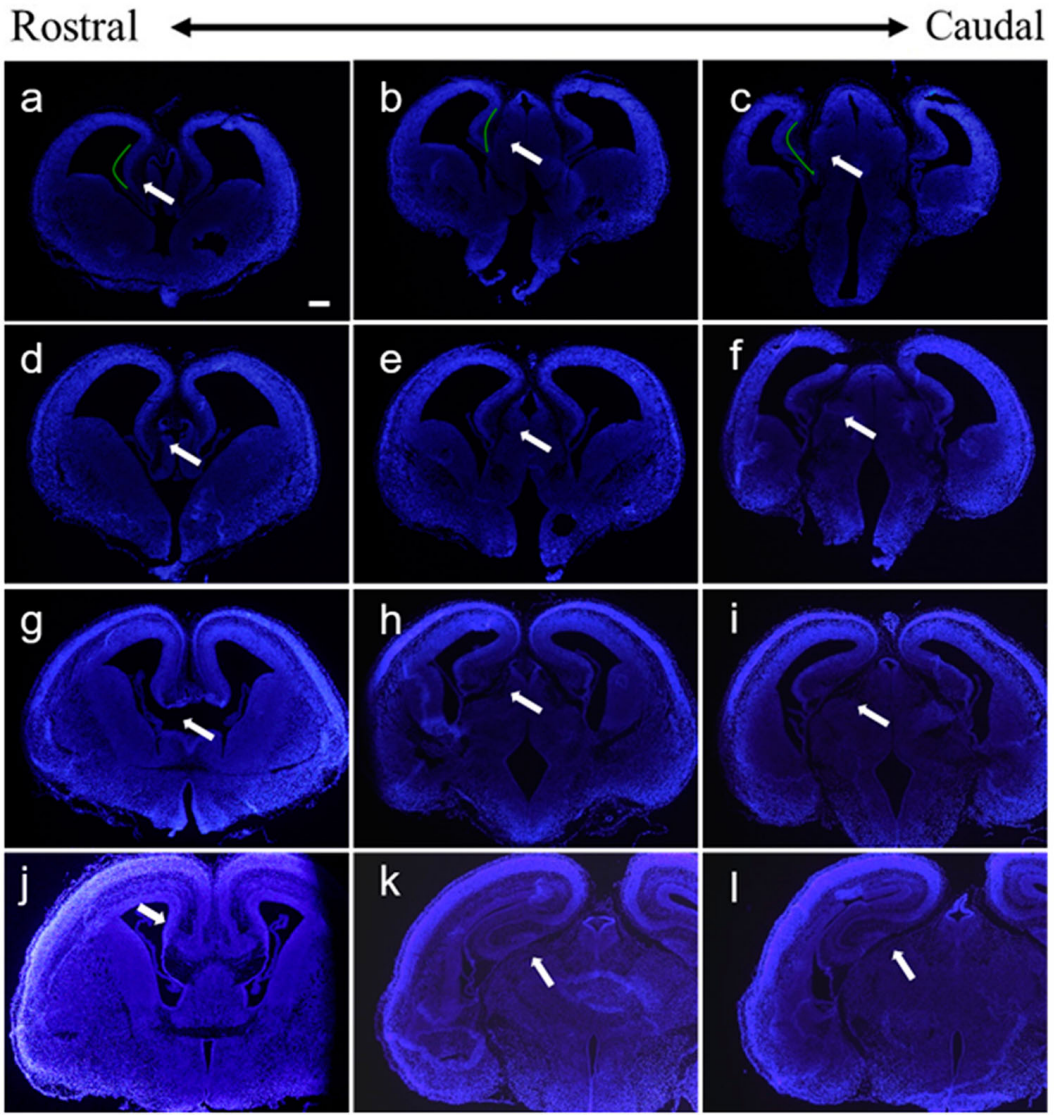
Coronal sections of the embryonic brain at different stages. (A–C) Hippocampus at E13.5 stage, (D–F) hippocampus at E14.5 stage, (G–I) hippocampus at E15.5 stage, and (J–L) hippocampus at E16.5 stage. The white arrows indicate the hippocampus at different stages. The scale bar was 200 μm.

Another major solution we provided in this study is to transfer plasmids into the hippocampus. In order to identify the suitable stage to operate IUE for the embryonic hippocampus, we stained hippocampus at different embryonic stages by immunohistochemistry. The results showed that hippocampus primordium was formed at E13.5 (Figure 5(A–C)), which only consisted of stratified periventricular neuroepithelial cells, and therefore, it is premature for electroporation. At E14.5, cornu ammonis has ultimately formed, the neuroepithelial cells layer was thickened, and the intermediate layer was formed (Figure 5(D–F)). At E15.5, dentate gyrus formed, and dentate gyrus folded with cornu ammonis which promote the formation of hippocampal fissure (Figure 5(G–I)). We found that the E14.5 brains were the most suitable for hippocampal transfection by IUE.

**Figure 6.**
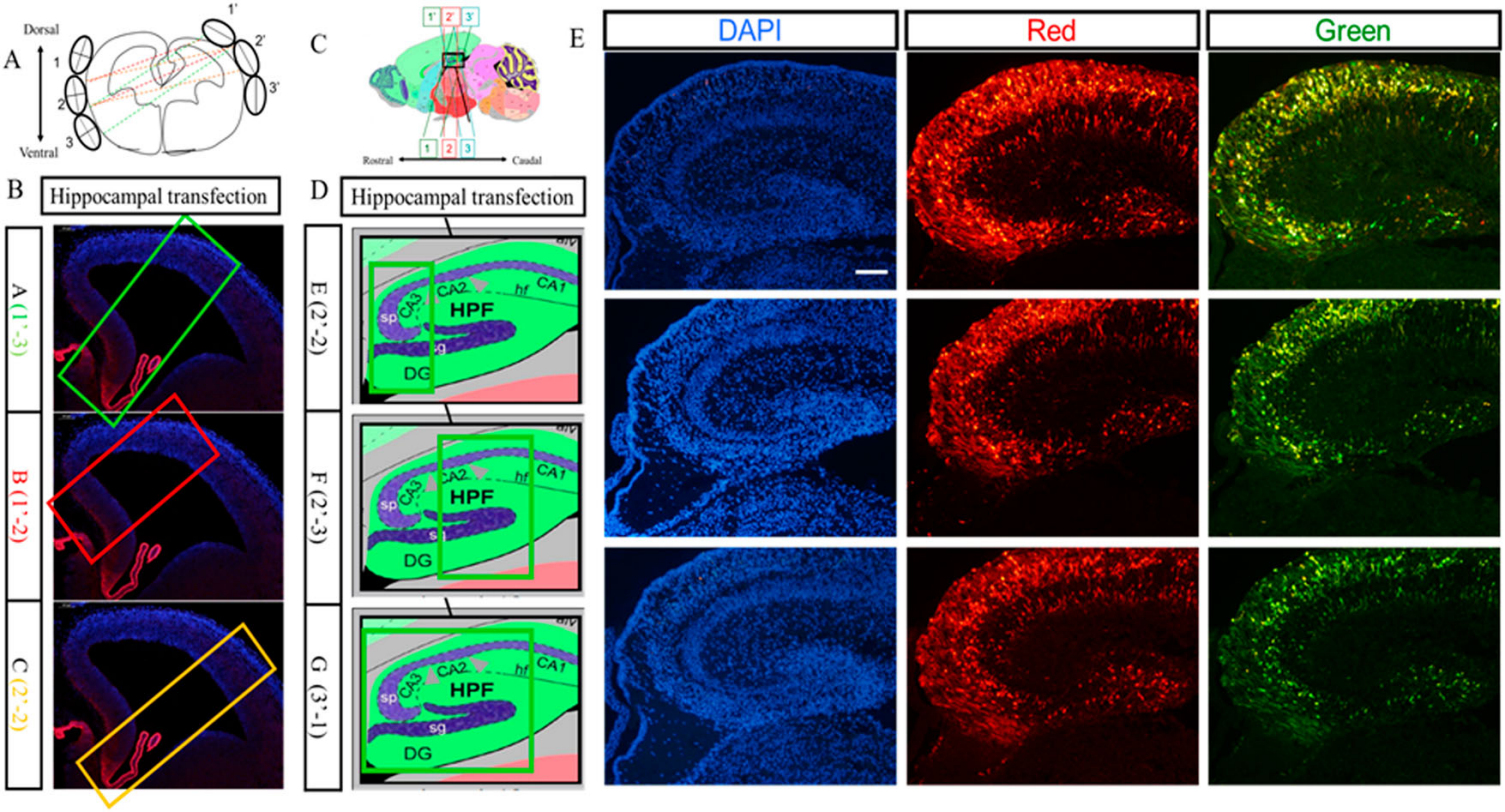
Transfection in the hippocampus region of the embryonic brain. (A) Combinations of different positions of electrodes at the coronal plane for transfection. (B) Transfected regions at the coronal plane resulting from different combinations of electrodes. (C) Combinations of different positions of electrodes at the sagittal plane for transfection. (D) Transfected regions at the sagittal plane resulting from different combinations of electrodes. (E) The hippocampus transfected with RFP and GFP vectors simultaneously. Two kinds of plasmids with RFP and GFP were transferred into hippocampus at E14.5 stage by IUE, and red fluorescence protein and green fluorescence proteins were expressed in the hippocampus region. The scale bar was 100 μm.

The hippocampus is a three-dimensional structure, so the position of the electrode appears to be particularly important to determine the area of electroporation, and we optimized here the electrode position to maximize the electroporation area. In Figure 6(A), the 1′ to 3′ represents the position of the positive pole of the electrode, and 1–3 represents the position of the negative pole. On the coronal section, electroporation region differed from the combination of electrodeposition, and the combination of position-1′ of the positive pole and position-3 of the negative pole was optimal as it covered most of the hippocampus region (Figure 6(B)). And the best combination for the sagittal section was position-3′ of the positive pole and position-1 of the negative pole, which also covered most of the hippocampus on the sagittal section (Figure 6(D)). The hippocampus was harvested at E18.5 and the sections of hippocampus showed that RFP and GFP could be detected expression simultaneously (Figure 6(E)).

## Discussion

The IUE has plenty of beneficiary effect; it also has limitations like surgical damages, transient transfection that produces false results. We described here the protocol and safety measures for IUE and how surgical damage can cause artificial ectopias and heterotopia in the developing cortex. We defined different markers to identify the micropipette puncture region that may produce an ectopias and heterotopia-like structures in the later stages of the development. Adaptation of these simple identification methods can distinguish between the real ectopias and heterotopia structure that created by the wound healing process of the needle puncture.

The tissue integrity was lost in the process of pseudo-ectopia and heterotopia formation. The needle punctured zone was seen to swell out at the periphery and at the ventricle zone of the cortex and the heterotopia and ectopia bulges could also be measured. To our knowledge, this DAPI staining would be a simple primary observation that can be helpful to identify these little structural deformities in developmental brains. In normal cases, the outer layered cortex used to be tuj1-positive (Halbach 2007), so the neuronal differentiation process in the brain could be measured by tuj1 staining. The improper differentiation was observed in the pseudo-heterotopia, and ectopia containing the brain. We observed an early differentiation induction at the damaged sites of brain. This early differentiation with the DAPI-bulges are a prominent marker for the pseudo-heterotopia and ectopia containing the brain (Figure 2).

To understand the distribution of neurons and their migration process, the morphological transition of the neurons at the IMZ region is important (Buchsbaum and Cappello 2019). The newborn neurons undergo for inside out pattern to reach at the CP. Therfore, the IMZ is an important area, where the neurons undergo morphological transitions. Then later, the bipolar-shaped neurons migrate upwards with the help of radial glial cells. For the brains with ectopia and heterotopia, we found the neuron’s distribution in the cortex was not uniformed. Later, we observed the structural integrity of the cortex was disturbed in the ectopic and heterotopic cortex. The marginal layer, the upper layer and the deep layer of the cortex were ruptured; also, overmigration of the neurons was also observed in the ectopic brains (Figure 3). While comparing with the ectopic brains, there were no significant changes in the number of proliferating cells, but the structure of the proliferating zone was changed with a bulged out erection in the surgically damaged brains. There was a significant increase in the number of KI67-positive cells at the upper layer region in the ectopic brains, implying the leakiness or disruption of the neuronal layers.

The solutions to avoid the artificial ectopia and heterotopia formation were described in the Result section. To avoid these situations, a proper injection technique should be followed and that would result in a suitable outcome for the researchers. Moreover, micro-injection in the hippocampus region would be another solution to avoid these unwanted circumstances. A description of suitable hippocampus injection was optimized and described here.

## Conclusions

The developmental neurobiology is an active area of research. IUE remains an important technique for gene transfer in the developing brains and that provides us the detail of different aspects in neuronal development, neuronal migration, and neuronal morphological transitions. Surgical damages during IUE injection may generate undesirable outcomes. Surgical damages during IUE procedure can create artificial ectopias and heterotopias in the developing cortex of mouse brain. Markers such as Ctip2, Laminin, and Reelin could identify surgical damage-related artificial ectopias and heterotopias in the developing cortex. Besides, we proposed the solutions to avoid surgical damages in IUE protocols. We hope this article may serve as a model for the identification of artificial ectopias and heterotopias in the developing mouse cortex in future studies.
